# The Promise of Infrared Spectroscopy in Liquid Biopsies for Solid Cancer Detection

**DOI:** 10.3390/diagnostics15030368

**Published:** 2025-02-04

**Authors:** Charlotte Delrue, Sander De Bruyne, Marijn M. Speeckaert

**Affiliations:** 1 Department of Nephrology, Ghent University Hospital, 9000 Ghent, Belgium; charlotte.delrue@ugent.be; 2 Department of Diagnostic Sciences, Ghent University, 9000 Ghent, Belgium; sanderr.debruyne@ugent.be; 3 Department of Laboratory Medicine, AZ Sint-Blasius, 9200 Dendermonde, Belgium; 4 Research Foundation-Flanders (FWO), 1000 Brussels, Belgium

**Keywords:** oncology, ATR-FTIR spectroscopy, machine learning

## Abstract

Attenuated total reflection-Fourier transform infrared (ATR-FTIR) spectroscopy has shown significant promise in the context of liquid biopsy, offering a potential tool for cancer diagnostics. Unlike traditional tissue biopsies, which may not fully capture the clonal heterogeneity of tumors, liquid biopsy reflects the dynamic state of the disease and its progression more comprehensively. Biofluids such as serum and plasma are low-cost, minimally invasive diagnostic media with well-established clinical uses. This review assesses the use of ATR-FTIR spectroscopy to detect biochemical changes in biofluids linked to various malignancies, including breast, ovarian, endometrial, prostate, bladder, kidney, pancreatic, colorectal, hepatic, esophageal, gastric, lung, and brain cancers. While ATR-FTIR offers the advantages of rapid, minimally invasive detection and real-time disease monitoring, its integration into clinical practice faces challenges, particularly in terms of reproducibility due to variability in sample preparation, spectral acquisition, and data processing. The translation of ATR-FTIR into routine diagnostics will require validation through large-scale cohort studies and multicenter trials to ensure its clinical reliability and effectiveness.

## 1. Introduction

Early detection of solid tumors is critical for improving patient outcomes, as the prognosis is significantly better when cancers are identified before symptoms appear. Moreover, ongoing advancements in molecular biology and cancer research have led to the development of targeted and effective therapies for various cancers. However, traditional diagnostic methods such as imaging and tissue biopsies can be invasive, costly, and may not effectively detect early stage cancers [1]. Liquid biopsy aims to identify easily accessible and operator-independent biomarkers for the detection of malignancies. Disease-related pathological changes often involve the disruption of both the concentration and conformation of biomolecules in the blood [2,3]. Over the past decade, liquid biopsy has emerged as a key tool in personalized medicine, allowing for the real-time monitoring of cancer progression and patient follow-up. Monitoring treatment response is critical for personalized cancer care as it supports survival by guiding adjustments to treatment plans. Despite improved therapies that enhance survival rates, recurrence remains a concern, and cancer survivors often experience lingering symptoms, side effects, and psychological stress related to their treatments. For both patients and physicians, the primary goal in follow-up care is to detect recurrences early. However, current follow-up methods fall short in this regard, as demonstrated in a meta-analysis of over 5000 patients, where only 40% of isolated locoregional recurrences were detected in asymptomatic individuals through routine examinations [4]. Liquid biopsy may be a highly effective and reliable tool for the real-time molecular profiling of patients [5]. Unlike traditional tissue biopsies, which may not fully capture the clonal heterogeneity of the tumor, liquid biopsy offers a more accurate reflection of the current state of the disease and the timing of its progression, including the impact of various internal and external factors during tumor development and metastasis [6]. The advantages of liquid biopsies include easy accessibility, suitability for both inpatients and outpatients, cost-effectiveness, ability to be collected sequentially and repeatedly for ongoing monitoring, and the capacity to detect changes in tumor mutation subtypes over time [7].

Liquid biopsy enables the extraction, enumeration, and molecular analysis of circulating tumor cells (CTCs), circulating tumor DNA (ctDNA), circulating microRNAs (miRNAs), and tumor-derived extracellular vesicles (tdEVs) from various body fluids, including peripheral blood, urine, cerebrospinal fluid (CSF), and pleural fluid. The real-time identification of predictive biomarkers through liquid biopsy holds significant promise to advance cancer therapy management [8]. tdEVs are nanoscale vesicles secreted by cancer cells that carry tumor-specific molecular cargo, making them valuable for noninvasive cancer diagnostics and monitoring. However, challenges such as EV heterogeneity, contamination during isolation, the lack of standardized protocols, and the difficulty in distinguishing tdEVs from non-tumor EVs hinder their clinical application [9,10]. Liquid biopsy is particularly focused on early cancer detection, enhanced staging, early relapse identification, real-time monitoring of therapeutic effectiveness, and the detection of therapeutic targets and resistance mechanisms [11]. However, these markers have limitations. The release of ct/circulating free (cf)DNA into the bloodstream is highly variable, and not all cancers or subtypes provide sufficient material for reliable detection. Early stage cancers, in particular, shed such low amounts of ctDNA that they are often beyond the detection capability of current techniques, akin to searching for a “needle in a haystack”. Moreover, the high cost of genetic-based methodologies limits their widespread use, especially for patients with non-specific symptoms, where the incidence of cancer is low. While tumor-derived signals are typically more abundant in late-stage cancer, signals from non-tumor-derived sources, such as the immune response, dominate in early stage disease. A pan-omics approach that combines both tumor- and non-tumor-derived signatures can address the intrinsic limitations of genetic-based liquid biopsies.

Transmission FTIR spectroscopy works by directly transmitting infrared beams through a sample. However, this approach can result in spectra that are strongly influenced by physical phenomena, such as light scattering, refraction, and dispersion. A more recent and simplified variation of this technology, known as attenuated total reflection-FTIR (ATR-FTIR) spectroscopy, effectively addresses these issues. ATR-FTIR spectroscopy involves directing the infrared beam through a high refractive index crystal, typically made of diamond, where it undergoes total internal reflection. This reflection creates an evanescent wave that penetrates slightly beyond the surface of the crystal into the sample placed on the top. The sample absorbs specific infrared wavelengths according to its molecular composition, leading to the attenuation of the IR beam. This attenuated beam is then detected after exiting the opposite end of the crystal [12].

The ATR mode, which can also use crystals such as germanium or zinc, exhibits optical properties different from those of transmission spectroscopy, thereby overcoming the spectral issues associated with the latter. For example, it reduces dispersion effects, leading to fewer spectral artifacts from light scattering during the analysis of biological samples [13]. This technique is highly suitable for clinical use because of its low cost, minimal sample preparation, and ability to provide rapid and reproducible results, making it an excellent tool for the routine monitoring of biofluids [14]. Serum is an ideal sample for ATR-FTIR analysis as it meets both critical conditions: its refractive index is lower than that of the crystal, ensuring internal reflectance, and its liquid nature allows for optimal contact with the crystal surface, facilitating the formation of a clear and stable evanescent wave within the 0.5–5 μm penetration depth. ATR crystals are available in various materials, including zinc selenide (ZnSe), zinc sulfide (ZnS), germanium (Ge), silicon (Si), and diamond. However, ZnSe, Ge, and diamond are most frequently used because of their favorable material [14].

ATR-FTIR spectroscopy analyses biochemical compositions within biological samples, detecting nucleic acids, proteins, lipids, and carbohydrates by identifying specific molecular conformations, bonding types, functional groups, and intermolecular interactions. Each molecule contributes to a characteristic spectrum based on the infrared radiation it absorbs, creating a composite spectral fingerprint that reflects the molecular composition and interactions within the genome, lipidome, proteome, and metabolome. Carbohydrates [9,15]. These spectral fingerprints reveal unique molecular alterations associated with specific diseases, thereby providing detailed diagnostic insights into a patient’s health status. The lipid region is characterized by several distinct peaks, including C=O stretching of ester linkages at approximately 1740 cm^−1^, symmetric C-H stretching around 2880 cm^−1^ (saturated fats), asymmetric C-H stretching around 2930 cm^−1^ (unsaturated fats), and C-H bending at approximately 1465 cm^−1^, which correspond to methylene scissoring modes in fatty acid chains. The protein region is defined by amide I bands, which correspond to C=O stretching, and amide II bands, which represent N-H bending and C-N stretching. These bands are key markers of the protein structure and provide insights into conformational changes in serum proteins. The nucleic acid region includes signals from the phosphate backbone of DNA and RNA, with PO₂^−^ asymmetric stretching occurring at approximately 1220 cm^−1^ and PO₂^−^ symmetric stretching at approximately 1080 cm^−1^. These peaks indicate nucleic acid integrity and may reflect disease-related changes in cellular turnover. Additionally, carbohydrates are identified by C-O stretching in the 1150–1000 cm^−1^ region, which highlights the presence of glycoconjugates and polysaccharides in the serum. Finally, the water bands absorbed in the 3550–3200 cm^−1^ region due to O-H stretching reflect hydration states, which can influence the overall spectral profile properties (Table 1) [14,16,17,18]. By quantifying vibrational modes and analyzing spectral data using machine learning, FTIR spectroscopy can be used to advance clinical diagnostics [17,19,20].

**Figure 1:**
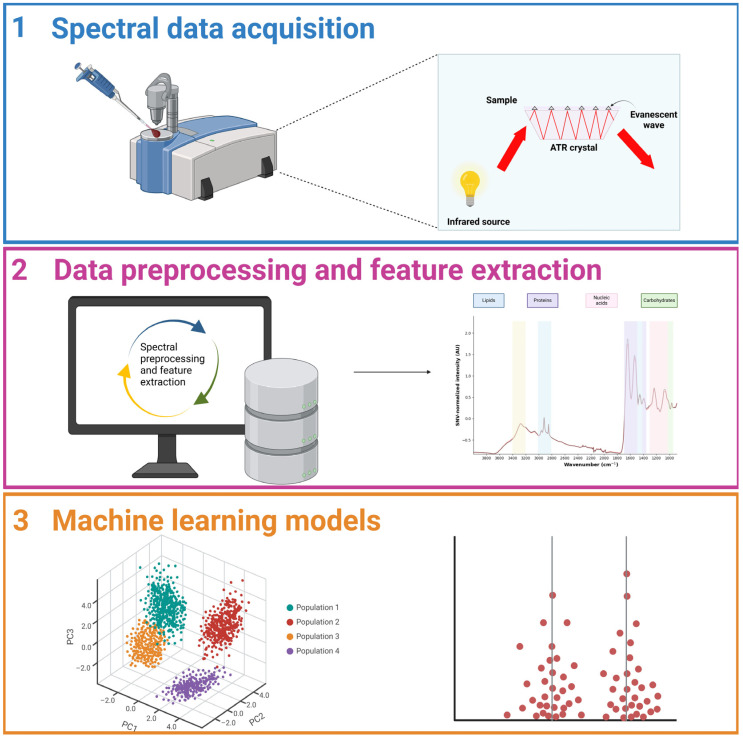
Workflow for solid cancer detection using ATR-FTIR spectroscopy and machine learning in liquid biopsies. In the first step, spectral data acquisition, liquid biofluid samples such as serum or plasma are applied to the ATR crystal of the spectrometer. Infrared light interacts with the sample through an evanescent wave formed at the crystal–sample interface, producing a unique spectral fingerprint that reveals the chemical makeup of the biofluid. In the second step, which includes feature extraction and data preparation, the acquired spectra are processed to reduce noise, correct baseline variations, and normalize the data. Important biochemical features of components such as proteins, carbohydrates, lipids, and nucleic acids are extracted for further investigation. During the machine learning stage, the processed spectral data are analyzed using methods like principal component analysis (PCA) or supervised models such as support vector machines (SVMs) to categorize samples into groups, including healthy, malignant, or specific cancer subtypes. Complementary to ATR-FTIR spectroscopy, Raman spectroscopy is another type of vibrational spectroscopy, which is currently being investigated in liquid biopsies. ATR-FTIR spectroscopy excels in its ability to provide rapid, cost-effective, and reproducible results with minimal sample preparation, making it well suited for clinical applications. However, its sensitivity can be limited, especially when detecting low-abundance biomarkers in complex biofluids. In contrast, Raman spectroscopy, particularly surface-enhanced Raman spectroscopy (SERS), is highly effective at identifying unique molecular fingerprints and detecting subtle molecular changes, even in low concentrations. While ATR-FTIR spectroscopy offers advantages in accessibility and clinical practicality, Raman spectroscopy outperforms in detecting low-abundance cancer biomarkers with greater precision. Together, these techniques present complementary strengths, suggesting potential synergy in cancer biomarker analysis [10].

Liquid biopsies are intrinsically complex since they contain a diverse range of biomolecules, such as proteins, lipids, nucleic acids, and metabolites. This complexity creates substantial analytical hurdles, necessitating ways for completely analyzing these components in a nondestructive and efficient manner. ATR-FTIR spectroscopy is well suited to addressing these difficulties for a variety of reasons. Firstly, ATR-FTIR spectroscopy is based on a nondestructive analysis, allowing for the direct characterization of liquid biopsy samples without the need for substantial preprocessing, hence preserving the sample’s native biochemical state [21]. Secondly, ATR-FTIR spectroscopy provides broad biochemical profiling by detecting and analyzing the vibrational signatures of multiple biomolecules simultaneously. This capability facilitates a holistic understanding of the sample’s molecular composition [22]. Furthermore, ATR-FTIR spectroscopy is quick and can perform high-throughput analysis, making it perfect for the clinical workflows required in liquid biopsy techniques. It also requires a modest sample volume, which is useful when working with limited biopsy material. Importantly, the ATR interface overcomes sample heterogeneity by normalizing the interaction of the sample with infrared light, yielding consistent and reproducible spectral data. To handle the complexities of liquid biopsy samples, ATR-FTIR is routinely combined with advanced data analysis techniques, like chemometrics and machine learning. These methods allow for the deconvolution of overlapping spectrum properties, resulting in the identification of specific biomarkers with high sensitivity and specificity, as seen in the early detection of various cancer kinds [23]. Figure 1 gives an overview of the workflow for solid cancer detection using ATR-FTIR spectroscopy and machine learning models in liquid biopsies.

In this review, we examine the evidence supporting the use of vibrational spectroscopy to generate spectral biomarkers in biofluids to detect different solid cancers in a rapid, minimally invasive, and cost-effective manner.

## 2. Breast Cancer

Breast cancer is one of the most commonly diagnosed cancers globally, consistently ranking among the top causes of cancer incidence and mortality. It accounts for a significant proportion of all cancer cases and deaths, being the leading cause of cancer diagnoses and fatalities among women in many countries. Breast cancer often holds the highest incidence rates in numerous regions and remains a major contributor to global cancer mortality, underscoring its impact on public health worldwide [24]. Early and accurate detection is critical for improving the outcomes. However, the current diagnostic landscape presents numerous challenges. Traditional screening methods, such as mammography and ultrasound, which are widely used, are subject to limitations, such as false positives, false negatives, and subjective interpretation. Biopsies, although the gold standard, are time-consuming and rely heavily on the pathologist’s expertise [25]. In this context, the potential of ATR-FTIR spectroscopy has emerged as a promising tool (Table 2).

Several major findings from a series of studies on breast cancer detection employing spectroscopic methods and machine learning models have been presented based on serum and plasma sample analyses. A study of 200 breast cancer patients and 459 non-cancerous controls found that key wavenumbers (2872 cm^−1^, 1261 cm^−1^, 1549 cm^−1^, 3351 cm^−1^, and 1025 cm^−1^) were crucial in discriminating between the two groups. In this study, the model’s threshold for classification was adjusted to optimize either sensitivity (to identify early stage cancers and minimize false negatives) or specificity (to prioritize high confidence in positive diagnoses and reduce false positives). The resulting model achieved an area under the curve (AUC) of 0.76, with 88% sensitivity at 43% specificity and 47% sensitivity at 87% specificity, depending on whether the model was sensitivity- or specificity-tuned. This adaptability demonstrates their findings’ practical application in a variety of diagnostic scenarios, such as screening asymptomatic populations or prioritizing symptomatic patients based on a healthcare system’s priorities [23]. However, this publication only describes ATR-FTIR, a method that works particularly well with liquid samples; it excludes critical information about preprocessing processes, which are required to ensure the accuracy and consistency of the results. A smaller study focused on the ratios of α-helix to β-pleated sheet proteins and amides II to III, yielding a sensitivity and specificity of 90% and 80%, respectively, with 100% sensitivity and 80% specificity for the latter ratio [26]. For plasma analyses, studies have also identified important wavenumbers that are excellent for distinguishing breast cancer patients from controls [22,24]. In a study of 56 breast cancer patients and 18 controls, wavenumbers related to DNA/RNA bands (1118–1052 cm^−1^) were identified as crucial for classification. The orthogonal partial least squares discriminant analysis (OPLS-DA) model developed from these data achieved perfect accuracy and a root mean square error of cross validation (RMSECV) below 0.005 across all molecular subtypes and control groups, with statistically significant differences observed between the groups. However, the small sample size and inclusion of only stages I-III may limit generalizability, given breast cancer’s molecular diversity. Furthermore, specific drying conditions were not standardized [27]. In a larger study with 476 breast cancer patients, successive projection algorithm-support vector machines (SPA-SVM) analysis highlighted several critical wavenumbers (1364 cm^−1^, 1277 cm^−1^, 1018 cm^−1^, 999 cm^−1^, 980 cm^−1^, 959 cm^−1^, and 901 cm^−1^), resulting in an AUC of 0.93, an accuracy of 92%, a sensitivity of 94%, and a specificity of 91%. This study demonstrated robust classification performance, with F-score and G-Score values of 93%, even in the face of data imbalance [28].

## 3. Ovarian Cancer

Ovarian cancer is a significant health concern, ranking among the leading causes of cancer-related deaths in women worldwide. While it is less common than some other cancers, it is particularly deadly due to challenges in early detection and diagnosis. Most cases are diagnosed in women over the age of 60 [24]. Currently, there are no standardized screening tests for ovarian cancer, highlighting the urgent need for new diagnostic tools, especially those capable of detecting the disease in its early stages, when treatment is most effective [29]. Current diagnostic methods such as pelvic examinations, transvaginal ultrasound, and serum CA-125 levels are often inadequate for early detection, leading to delayed diagnosis and poor prognosis. These tools suffer from limitations such as low sensitivity and specificity, particularly in early stage disease, and are often influenced by non-cancerous conditions, which complicate diagnosis [30]. The gold standard histopathological examination following biopsy is invasive and time-consuming.

In studies examining ovarian cancer diagnostics using ATR-FTIR spectroscopy on serum samples, significant findings have been reported. One study involving 125 ovarian cancer patients and 459 non-cancerous controls identified critical wavenumbers (1529 cm^−1^, 3327 cm^−1^, 3244 cm^−1^, 1263 cm^−1^, and 1084 cm^−1^) that played a key role in distinguishing between the groups. The resulting model achieved an AUC of 0.86, with a sensitivity of 95% at 42% specificity and 46% at 95% specificity, depending on the tuning of the model [23]. A study involving 30 ovarian cancer patients and 30 endometrial non-cancer controls analyzed serum samples and identified specific wavenumbers related to molecular structures, such as 1030 cm^−1^ for C-O ribose stretching and 1585 cm^−1^ for amide I. Using feature selection and the k-nearest neighbors algorithm (KNN), this analysis achieved a classification rate of 95%. In plasma samples from the same cohort, the study applied feature selection-least absolute shrinkage and selection operator (FS-LASSO) followed by eClass1 as the classification method, achieving an even higher classification rate. Key wavenumbers important for classification included 1034 cm^−1^ (collagen), 1072 cm^−1^ (nucleic acid band), 1115 cm^−1^ (RNA), 1377 cm^−1^ (oligonucleotide base and sugar), 1412 cm^−1^ (C-N stretching and N-H and C-H deformation), and 1589 cm^−1^ (ring C-C stretch of phenyl) [31].

ATR-FTIR spectroscopy also holds significant promise for the precise classification, staging, and grading of cancer. In a notable study, Lima et al. [32] achieved sensitivity and specificity rates as high as 100% for ovarian cancer diagnosis by integrating ATR-FTIR spectroscopy with techniques such as the successive projection algorithm and genetic algorithm coupled with linear discriminant analysis (GA-LDA). Their research demonstrated the accuracy of the method in identifying ovarian cancer stages, histological types, and age-related differences through plasma and serum analysis, underscoring its potential as a robust tool for biomarker discovery and population-based ovarian cancer screening, but the absence of validation in larger, independent cohorts raises concerns.

## 4. Endometrial Cancer

Endometrial cancer, the most common cancer of the female reproductive organs, primarily affects postmenopausal women, with incidence increasing with age. In 2024, it was estimated that approximately 67,880 new cases of uterine cancer, including endometrial cancer, would be diagnosed, and around 13,250 deaths would occur. Advances in early detection and treatment have improved outcomes for many, but rising obesity rates, a significant risk factor, contribute to increasing incidence in some populations. Despite progress, disparities in survival exist [24]. Diagnosis typically depends on histological examination of an endometrial tissue sample. However, this invasive procedure is usually reserved for patients who show signs of endometrial pathology or have a thickened endometrium detected on transvaginal ultrasound. The poor specificity, approximately 51.5% at an endometrial thickness of 5 mm in postmenopausal women, results in a large number of women needing further testing before significant endometrial pathology can be excluded [33]. In a study analyzing blood samples from 30 endometrial cancer patients and 30 non-cancer controls, both plasma and serum samples were examined for specific molecular signatures using ATR-FTIR spectroscopy. Key wavenumbers identified in the plasma samples included 1026 cm^−1^ (glycogen), 1080 cm^−1^ (νsPO_2_^−^ related to RNA), 1408 cm^−1^ (COO- symmetric stretching of fatty acids and amino acids), 1589 cm^−1^ (ring C-C stretch of phenyl), and 1744 cm^−1^ (lipids), leading to a highest classification rate of 77.08% ± 15.87% using feature selection techniques like feature selected-Fisher, followed by KNN and Identity classifiers. For the serum samples, wavenumbers such as 1026 cm^−1^ (glycogen), 1080 cm^−1^ (νsPO_2_^−^), 1153 cm^−1^, 1466 cm^−1^, 1520 cm^−1^, and 1744 cm^−1^ (lipids) were identified, with an ensemble of feature selection-Fisher, FS-LASSO, feature selection, and SVM achieving a classification rate of 81.67% ± 18.34% [31].

## 5. Prostate Cancer

Prostate cancer is one of the most commonly diagnosed cancers globally and represents a significant public health challenge due to its high incidence and mortality rates, particularly in older men. It ranks as the second most frequent cancer and the fifth leading cause of cancer death among men worldwide [24]. However, prostate-specific antigen (PSA) levels can be elevated in some men without cancer [34], and factors such as urinary tract infections, medications, and surgery can also affect PSA levels [35]. Consequently, tissue biopsy remains the gold standard for confirming prostate cancer, as PSA screening alone may not be clinically reliable. The variability in PSA screening guidelines has led to overtesting and unnecessary biopsies, raising concerns in healthcare practice [36]. Therefore, there is an urgent need for improved screening tools to reduce unnecessary invasive procedures and better protect patient well-being. A study exploring the use of ATR-FTIR spectroscopy for diagnosing prostate cancer tested various biological samples and diagnostic methods to identify important molecular markers and to evaluate how accurately they could classify the disease. Among serum samples, a study involving 199 prostate cancer patients and 459 non-cancerous controls identified significant wavenumbers at ~1357 cm^−1^ and ~2947 cm^−1^. The model demonstrated an AUC of 0.86, with 95% sensitivity at 44% specificity and 45% sensitivity at 96% specificity for sensitivity- and specificity-tuned models, respectively [23]. For plasma samples, a study involving 43 prostate cancer patients and 33 healthy controls identified several key molecular markers, including tyrosine (830 cm^−1^ and 850 cm^−1^), DNA/RNA (940 cm^−1^, 1085 cm^−1^, 1340 cm^−1^, and 1420 cm^−1^), β-carotene (1160 cm^−1^ and 1525 cm^−1^), and various lipid and protein markers (622 cm^−1^, 644 cm^−1^, 1300 cm^−1^, 1400–1470 cm^−1^, and 1640–1670 cm^−1^). A partial least squares-discriminant analysis (PLS-DA) model was able to successfully discriminate between patients with prostate cancer and healthy controls, achieving sensitivity and specificity rates between 90% and 99%. Classification fitting analysis further highlighted the involvement of key analytes in the development and progression of prostate cancer [37]. However, caution is required when interpreting the performance metrics of an OPLS-DA model applied to a limited dataset, as small sample sizes can lead to overfitting and inflated accuracy estimates, reducing the generalizability of the findings. In another study using saliva samples from 17 prostate cancer patients and healthy controls, only small differences were observed around 1500–1650 cm^−1^ and 1000–1100 cm^−1^, with a principal component analysis (PCA) followed by a quadratic discriminant analysis (PCA-QDA) model achieving 93% testing accuracy, 100% clinical sensitivity, and 92% specificity using five principal components (84.2% explained variance) [38]. However, these results have to be interpreted with caution because this study introduces variability due to uncontrolled hydration levels in saliva samples, which impacts spectral reproducibility and diagnostic accuracy. Few studies investigated the role of plasma and serum EVs in prostate cancer. The ATR-FTIR analysis in four prostate cancer patients vs. eight controls (four healthy controls and four with benign prostate hyperplasia) also detected key wavenumbers, including 1656 cm^−1^ (amide I and alpha-helix structures), 1544 cm^−1^ (amide II), 1620–1640 cm^−1^ and 1670–1695 cm^−1^ (beta-sheet structures), and 3298 cm^−1^ (amide A) [39]. Another study combined ATR-FTIR analysis of urinary EVs with a PCA-LDA statistical model as an innovative approach for noninvasive early detection of prostate cancer. The spectral differences observed between EVs from prostate cancer patients and healthy individuals, along with the performance of the LDA-derived classifier, which achieved a sensitivity of 83.33% and specificity of 60%, highlight the potential of the ATR-FTIR technique as a point-of-care diagnostic tool for prostate cancer using urine samples [40]. However, the development of standardized methodologies to isolate specific EV subtypes could further enhance biomarker identification and improve diagnostic accuracy.

## 6. Pancreatic Cancer

Pancreatic cancer, characterized by poor prognosis and high mortality, accounted for an estimated 511,000 new cases and 467,000 deaths worldwide in 2022, ranking as the 12th most common and 7th deadliest cancer globally. Smoking, obesity, type 2 diabetes, and alcohol consumption are key modifiable risk factors, while genetic predisposition plays a smaller role. Mortality remains high due to late-stage diagnoses and limited treatment options, with survival among the lowest of all cancers. Efforts to reduce risk factors and improve early detection are critical as pancreatic cancer’s global burden continues to rise [24]. Currently, early diagnostic methods for pancreatic cancer in the clinical setting include computed tomography, magnetic resonance imaging, endoscopic ultrasonography, endoscopic retrograde cholangiopancreatography, and magnetic resonance cholangiopancreatography [41]. Biomarkers are also playing an increasingly important role in the early detection and screening of high-risk individuals. Although carbohydrate antigen 19-9 (CA19-9), carcinoembryonic antigen, and CA125 are the most commonly used biomarkers for pancreatic cancer, their accuracy in early diagnosis remains suboptimal, with CA19-9 showing a sensitivity of 79–81% and specificity of 82–90% in symptomatic patients [42,43]. Consequently, many research teams are actively exploring and developing new biomarkers for early diagnosis. Among the promising areas of research, liquid biopsy has emerged as a potential tool for identifying biomarkers for early detection of pancreatic cancer [44].

In studies investigating the potential of ATR-FTIR spectroscopy and machine learning algorithms for detecting pancreatic cancer, serum samples were analyzed from different cohorts of patients. In one study, serum samples from 166 patients with pancreatic cancer and 459 non-cancer subjects were analyzed using specific wavenumbers (3277 cm^−1^, 1529 cm^−1^, 1636 cm^−1^, 1288 cm^−1^, and 2784 cm^−1^). The resulting diagnostic model demonstrated an AUC of 0.84. When the model was tuned for sensitivity, it achieved 95% sensitivity and 42% specificity. Conversely, when optimized for specificity, it reached 93% specificity with 44% sensitivity [23]. In another study involving 100 patients with pancreatic cancer, 100 healthy controls, and 35 symptomatic controls, serum samples were analyzed at wavenumbers of approximately 1570 and 1500 cm^−1^, 1270 cm^−1^, and 1000–1100 cm^−1^. Using machine learning algorithms such as RF, PLS-DA, and SVM, researchers achieved a sensitivity of up to 92% and a specificity of 88% when distinguishing between pancreatic cancer patients and healthy controls, with an AUC of 0.95. Additionally, when comparing pancreatic cancer cases to symptomatic controls, the model produced a balanced sensitivity and specificity above 75%, along with an AUC of 0.83 [21]. These findings suggest that blood serum analysis combined with advanced modeling techniques holds promise for improving early detection of pancreatic cancer.

## 7. Colorectal Cancer

Colorectal cancer (CRC) ranks as the third most commonly diagnosed cancer globally. Incidence rates are substantially higher in high-Human Development Index (HDI) countries compared to low-HDI settings, reflecting lifestyle, dietary patterns, and access to screening. In high-HDI countries, CRC incidence rates have stabilized or declined, partly due to improved screening and dietary changes. Conversely, incidence is rising in transitioning countries, driven by urbanization, dietary shifts toward higher meat and fat consumption, increasing obesity, and sedentary lifestyles. Despite these trends, mortality rates are decreasing in higher-HDI settings because of early detection and advancements in treatment, while they remain high in lower-HDI regions due to limited healthcare access and late-stage diagnosis [24]. Colonoscopy and the fecal immunochemical test (FIT) are widely recognized as essential tools for early colorectal cancer detection [45]. Colonoscopy allows for the identification and removal of early precancerous lesions by a clinician, but it is an invasive and costly procedure with low patient compliance [46]. As an alternative, FIT is a noninvasive, cost-effective test commonly used for screening average-risk populations, particularly those who decline colonoscopy. However, FIT has only modest accuracy, with sensitivities below 70% for detecting colorectal cancer and under 50% for detecting advanced adenoma [3]. Consequently, colorectal cancer screening faces challenges owing to low adherence rates (e.g., colonoscopy) and suboptimal detection rates (e.g., FIT). In studies focusing on the detection of colorectal cancer by ATR-FTIR spectroscopy using serum samples, significant results have been achieved through the analysis of specific wavenumbers and spectral shifts. In one study, serum samples from 73 patients with colorectal cancer and 44 healthy controls were analyzed, revealing a redshift of 2 cm^−1^ in the amide I band and a significant redshift of 10 cm^−1^ in the amide II band. PLS-DA demonstrated a high AUC of 0.985, indicating a strong performance in distinguishing colorectal cancer patients from healthy individuals [22]. However, these results need to be interpreted with caution due to class imbalance, the small cohort size, and the need for broader clinical validation to confirm diagnostic robustness. Another study involving 200 colorectal cancer patients and 459 non-cancerous subjects analyzed serum samples at wavenumbers of 1530 cm^−1^, 3351 cm^−1^, 3234 cm^−1^, 1246 cm^−1^, and 1666 cm^−1^. The resulting model also showed robust performance, with an AUC of 0.91. When the model was tuned for sensitivity, it achieved 97% sensitivity and 44% specificity. Conversely, when optimized for specificity, the model achieved 97% specificity and 45% sensitivity [23].

## 8. Hepatocellular Cancer

Hepatocellular carcinoma (HCC), the most common primary liver cancer, accounts for 75–85% of liver cancer cases globally and ranks as the sixth most common cancer and third leading cause of cancer-related deaths. Key risk factors include HBV and HCV infections, obesity, type 2 diabetes, non-alcoholic fatty liver disease (NAFLD), alcohol use, smoking, and aflatoxin exposure in contaminated food. HCC’s prognosis is poor, with a 5-year survival rate below 20%, emphasizing the need for enhanced prevention through vaccination, antiviral therapies, and lifestyle modifications [24]. Currently, HCC screening relies on liver ultrasonography. However, its sensitivity for detecting early stage HCC in obese patients is only 23%. Alternative diagnostic tools for HCC include biochemical tests that measure liver enzyme levels and α-fetoprotein (AFP), which is the only widely recognized blood-based biomarker for HCC. The sensitivity of AFP is limited (approximately 60%, depending on the threshold value), and both the American and European Association for the Study of the Liver do not routinely recommend AFP as a screening biomarker for HCC. However, no biomarkers studied thus far have proven to be sufficiently effective for screening [47,48,49,50].

In studies involving plasma samples, vibrational spectroscopy has shown promising results in distinguishing HCC patients from cirrhotic patients and healthy controls. In one study comparing 20 obese cirrhotic patients with HCC to 17 without HCC, IR focused on amide I (1643 cm^−1^) and amide II (1547 cm^−1^) bands achieved a sensitivity of 0.780 and specificity of 0.905 using PLS-DA [47]. Another study involving 40 HCC patients and 60 cirrhotic patients identified key spectral features at 1649 cm^−1^, 1639 cm^−1^, and 1547 cm^−1^, with the FTIR method yielding an AUC of 0.83, an accuracy of 0.756, a sensitivity of 0.770, and a specificity of 0.748 using RF analysis [51].

For serum samples, significant differences were observed in spectral features when comparing patients with HCC to healthy controls. In one study of 25 HCC subjects versus 44 healthy controls, a redshift in the amide I band by 2 cm^−1^ was detected, resulting in an AUC of 0.987 using PLS-DA [22]. Another study comparing 39 patients with HCC to 56 healthy individuals found that an analysis in the lipid range of 2900–2800 cm^−1^ using PLS-DA achieved a sensitivity of 92.85% and a specificity of 95.23% [52]. In a study investigating 20 HCC, 60 cholangiocarcinoma (CCA), 20 biliary disease (BD), and 50 healthy controls, a neural network achieved 80–100% sensitivity and 83–100% specificity for differentiating CCA from healthy controls based on the spectral region 3000–2800 + 1800–1000 cm^−1^. Due to overlapping spectral signatures, it was difficult to differentiate CCA from HCC and BD [53].

Finally, a pilot study, involving 20 non-viral HCC patients and 19 controls, demonstrated that PCA-LDA-assisted ATR-FTIR spectral biomarkers on tdEVs achieved high diagnostic accuracy (AUC of 0.91) and outperformed traditional HCC markers, despite limitations such as small sample size and potential EV isolation impurities [9].

## 9. Esophageal and Gastric Cancer

Esophageal and gastric cancers collectively contribute significantly to the global cancer burden, with esophageal cancer ranking as the 11th most commonly diagnosed cancer and the 7th leading cause of cancer death, and gastric cancer as the 5th most diagnosed cancer and 5th in cancer mortality globally in 2022. Both cancers exhibit marked male predominance and poorer survival outcomes due to late-stage diagnoses, emphasizing the need for preventive strategies such as infection control and lifestyle interventions [24]. The diagnosis of esophagogastric cancers typically involves obtaining mucosal samples through endoscopy, which are then analyzed by a skilled pathologist [54]. A study investigating ATR-FTIR spectroscopy combined with variable selection methods, such as successive projections or genetic algorithms (GAs), and integrated with quadratic discriminant analysis (QDA), successfully identified spectral biomarkers in biofluids for accurately diagnosing different stages of esophageal adenocarcinoma (OAC) in a clinical hospital setting. In the case of esophageal cancer, involving a cohort of patients with various stages of the disease (including normal, inflammatory, Barrett’s oesophagus, low-grade dysplasia (LGD), high-grade dysplasia (HGD), and esophageal adenocarcinoma), the genetic algorithm followed by quadratic discriminant analysis (GA-QDA) model using serum spectra achieved 100% accuracy, sensitivity, specificity, and F-scores across all esophageal adenocarcinoma stages. The SPA-QDA model, which used seven selected variables, and the PCA-QDA model, which utilized seven principal components covering more than 90% of data variance, both demonstrated sensitivities and specificities exceeding 71% for the normal, inflammatory, Barrett’s, and esophageal adenocarcinoma stages. Regarding plasma samples, the same study on esophageal cancer also found a robust classification performance. The GA-QDA model, based on 15 selected wavenumbers, achieved nearly perfect classification with only a few errors in both the training and validation sets. Similarly, the PCA-QDA model, which uses seven principal components, and the SPA-QDA model for LGD classification also achieved 100% accuracy, sensitivity, specificity, and F-scores for the normal, inflammatory, Barrett’s, and esophageal adenocarcinoma classes. These results underscore the effectiveness of vibrational spectroscopy in differentiating between various stages and conditions associated with esophageal cancer [55].

## 10. Lung Cancer

Lung cancer is the leading cause of cancer deaths worldwide, with 2.5 million new cases and 1.8 million deaths in 2022, accounting for 12.4% of all cancers and 18.7% of cancer deaths. Smoking is the primary risk factor, shaping global and regional trends, with variations reflecting different stages of the tobacco epidemic across populations. Environmental factors, such as air pollution and occupational exposures, also contribute, particularly among non-smokers [24]. Owing to the high incidence and mortality associated with lung cancer, early detection offers substantial benefits, including improved patient outcomes and reduced healthcare costs [56]. However, the widespread use of low-dose computed tomography for lung cancer screening in high-risk populations presents a notable challenge. One significant drawback of low-dose computed tomography is the high rate of false-positive results, which limits its effectiveness as a screening tool. Additionally, the potential radiation risks associated with repeated annual low-dose computed tomography screenings have not been fully addressed. While low-dose computed tomography is highly sensitive and commonly recommended for lung cancer detection, identifying hidden tumors in the lung requires higher specificity and more advanced techniques [57]. Therefore, integrating well-validated liquid biopsy biomarkers into clinical prediction models is essential for enhancing early diagnosis and risk assessment, particularly for monitoring small lung nodules [58]. Moreover, these biomarkers may play a critical role in improving the prognosis of lung cancer and advancing precision medicine in its treatment [59].

In recent studies on lung cancer detection using biospectroscopy, significant findings have been reported in both saliva and blood samples. For saliva samples, one study involving 31 patients with lung cancer and 950 healthy controls identified small spectral differences in the 1650–1500 cm^−1^ and 1100–1000 cm^−1^ regions. Using a PCA-QDA model with eight principal components, which explained 90% of the variance, the study achieved a testing accuracy of 91% with 100% clinical sensitivity and 91% specificity. Saliva, as a diagnostic medium, introduces variability due to hydration levels, which were not controlled in this study [38]. However, the study introduces variability due to uncontrolled hydration levels in saliva samples, which impacts spectral reproducibility and diagnostic accuracy.

In one study investigating serum samples of 201 lung cancer patients and 459 non-cancerous subjects, the diagnostic model identified spectral features at 1167 cm^−1^, 1074 cm^−1^, 1532 cm^−1^, 2750 cm^−1^, and 1124 cm^−1^. The model achieved an AUC of 0.91, with a perfect sensitivity of 100% at a specificity of 40% and a sensitivity of 47% at a specificity of 95%, depending on whether the model was tuned for sensitivity or specificity [23].

## 11. Brain Cancer

Brain cancer, encompassing primary and metastatic tumors, accounted for 321,000 new cases and 248,000 deaths globally in 2022. Men are slightly more affected than women, and gliomas, including aggressive glioblastomas (GBMs), dominate adult cases, while childhood cases often involve medulloblastomas. Known risk factors include genetic predisposition, high-dose radiation exposure, and hereditary syndromes, although most cases lack a clear cause. Survival rates remain low, especially for GBMs, despite advances in molecular profiling and targeted therapies [24]. Currently, the diagnosis of GBMs primarily relies on neuroimaging techniques, along with histopathological and molecular analyses of tissues obtained through resection or biopsy. While these methods have their own benefits, they also present significant limitations [60]. Magnetic resonance imaging (MRI), which is essential for the initial diagnosis and anatomical characterization of GBMs, is unable to differentiate GBMs from other brain diseases and concurrent pathological processes. Moreover, MRI features often fail to accurately correlate with the molecular characteristics of the tumor, and it can be challenging to distinguish actual tumor recurrence from pseudoprogression [61]. Tissue biopsy, another common diagnostic approach, allows for detailed histological and molecular analyses but has significant drawbacks. The procedure is highly invasive and carries inherent risks that make repeated sampling difficult. Additionally, a biopsy may not fully capture a tumor’s intratumoral heterogeneity and cannot provide real-time insight into tumor activity [62]. In response to these challenges, liquid biopsy has emerged as a promising noninvasive diagnostic tool. This approach detects and quantifies tumor-derived materials present in biofluids and offers a valuable complement to traditional diagnostic techniques. Recently, genomics, circulating tumor cells, and extracellular vesicles have also been studied. However, ctDNA is specific but limited by its short half-life and its release from only a subset of tumor cells [63]. MiRNAs lack specificity and standardized extraction methods [64]. CTCs are informative but scarce and challenging to isolate [64]. Extracellular vesicles carry valuable cargo, but their diagnostic use is hindered by the absence of standardized methods for isolating extracellular vesicles, coupled with their release by non-neoplastic cells, resulting in non-tumoral extracellular vesicles in the blood [65]. A promising alternative for liquid biopsy is the application of ATR-FTIR spectroscopy to blood samples. In recent studies focusing on the detection of brain cancer using serum samples, ATR-FTIR spectroscopy has shown promising results. One study involving 247 patients with brain cancer and 459 non-cancerous subjects identified important spectral features at 1523 cm^−1^, 1607 cm^−1^, 3278 cm^−1^, 2861 cm^−1^, and 1256 cm^−1^ for classification. This model achieved an AUC of 0.90, with a sensitivity of 95% at a specificity of 42% and a sensitivity of 46% at a specificity of 99%, depending on whether the model was tuned for sensitivity or specificity [23]. This study is limited by the lack of tumor-specific control groups, the absence of genetic tumor information, and reliance on retrospective data requiring further prospective validation. Another study of 90 brain cancer patients and 87 control patients who showed no symptomatic indications of cancer used a PLS-DA model. For the T1 cohort, the PLS-DA model achieved a sensitivity of 98.5% and a specificity of 95.1%, with a balanced accuracy of 96.8%. For the T2/FLAIR cohort, the model showed a sensitivity of 88.7%, specificity of 94.7%, and balanced accuracy of 91.7% [66].

However, the reduced discriminatory performance following filtration, the dependence on high-molecular-weight components for accurate stratification, and the necessity of larger cohorts to validate clinical applicability must be carefully considered before accepting these results as definitive. A larger study involving 487 brain tumor samples and 237 healthy controls also used blood serum samples. This study reported the spectral features at various wavelengths and compared three different machine learning techniques: PLS-DA, RF, and SVM. The PLS-DA model achieved a sensitivity of 90.5% and a specificity of 91.1% in distinguishing patients with brain cancer from healthy controls. Despite the low prevalence of brain tumors (1.6%), the PLS-DA model yielded a positive predictive value of 14.2%, demonstrating that these machine learning approaches, with balanced accuracies of approximately 90%, are cost-effective for brain cancer detection [67].

**Table 2:** Overview of the results in multiple cancers of ATR-FTIR spectroscopy on serum, plasma, salivary, and urinary samples.

Cancer	Study Population	Matrix	Wavenumbers	Machine Learning Model	Performance Metrics	Limitations	Ref.
Breast cancer	200 patients with breast cancer vs. 459 NCS	Serum	2872 cm^−1^, 1261 cm^−1^, 1549 cm^−1^, 3351 cm^−1^, and 1025 cm^−1^	NS	The breast cancer model yielded an AUC of 0.76, showing 88% sensitivity at 43% specificity and 47% sensitivity at 87% specificity for the sensitivity- and specificity-tuned model, respectively.	Limited methodological clarity regarding preprocessing and the lack of tumor-specific control groups, the absence of genetic tumor information, and the reliance on retrospective data.	[23]
	56 patients with breast cancer vs. 18 controls	Plasma	1118–1052 cm^−1^ (predominantly DNA/RNA bands)	OPLS-DA	The OPLS-DA model achieved perfect accuracy and an RMSECV below 0.005 for all molecular subtypes and the control group. The wavenumbers with the highest iPCA peaks (1117 cm^−1^, 1089 cm^−1^, 1081 cm^−1^, 1075 cm^−1^, 1057 cm^−1^, and 1052 cm^−1^) were used in a MANOVA, showing significant differences (Wilks’ Lambda, *p* < 0.001) between the molecular subtypes and the control.	The small sample size and inclusion of only stages I–III breast cancer limit the generalizability given breast cancer’s molecular diversity.	[27]
	10 patients with breast cancer vs. 10 healthy controls	Serum	1700–1600 cm^−1^, 1580–1480 cm^−1^, and 1140–1000 cm^−1^	PCA-DA	The sensitivity and specificity of the ratio of α-helix to β-pleated sheet in proteins were both 90%. Similarly, the ratio of amide II to amide III (I1556/I1295) achieved a sensitivity of 100% and a specificity of 80%.	The study’s small cohort size reduces the statistical power and the robustness of the reported diagnostic performance metrics.	[26]
	66 patients with breast cancer vs. 80 healthy controls	Serum	1306–1250 cm^−1^	PCR	The PCR method achieved a sensitivity of 92.3% and a specificity of 87.1%, comparable to the performance of mammography and ultrasound.	The study does not clarify how its spectral findings compare to other diagnostic modalities, limiting broader clinical utility.	[68]
	476 patients	Plasma	1364 cm^−1^, 1277 cm^−1^, 1018 cm^−1^, 999 cm^−1^, 980 cm^−1^, 959 cm^−1^, and 901 cm^−1^	SPA-SVM	SPA-SVM achieved the highest classification performance with an AUC of 0.929, an accuracy of 92.9%, a sensitivity of 94%, and a specificity of 91% in detecting breast cancer samples based on an external test set (15% of samples, *n* = 71 patients). Approximately 70% of samples (*n* = 334 patients) were used for model construction, and another 15% were used for internal validation (*n* = 71 patients). The overall classification performance, as indicated by F-score and G-Score values of 93%, demonstrated robustness regardless of data imbalance.	The lack of longitudinal validation to confirm the technique’s effectiveness over time, the absence of external validation across diverse populations, the potential variability introduced by patient-specific factors (e.g., comorbidities or metabolic differences), the limited focus on a single cancer type without comparison to other malignancies or benign conditions, and the challenges in translating laboratory-controlled protocols to clinical settings where sample handling and variability may differ significantly.	[28]
Ovarian cancer	125 patients with ovarian cancer vs. 459 NCS	Serum	1529 cm^−1^, 3327 cm^−1^, 3244 cm^−1^, 1263 cm^−1^, and 1084 cm^−1^	NS	The ovarian cancer model achieved an AUC of 0.86, with a sensitivity of 95% at a specificity of 42% and a sensitivity of 46% at 95% specificity for the sensitivity- and specificity-tuned models, respectively.	Limited methodological clarity regarding preprocessing and the lack of tumor-specific control groups, the absence of genetic tumor information, and the reliance on retrospective data.	[23]
	Ovarian cancer cases (*n* = 30) and endometrial NCS (*n* = 30)	Plasma	1034 cm^−1^ (collagen), 1072 cm^−1^ (nucleic acid band), 1115 cm^−1^ (RNA), 1377 cm^−1^ (oligonucleotide base and sugar), 1412 cm^−1^ (stretching C-N, deformation N-H, and deformation C-H), and 1589 cm^−1^ (ring C-C stretch of phenyl)	FS-LASSO followed by eClass1	The best classification system achieved a classification rate of 96.67% ± 7.03%.	Small sample size, the lack of external validation across diverse populations, and potential age-related spectral variability between cancer and control groups.	[31]
	Ovarian cancer cases (*n* = 30) and endometrial NCS (*n* = 30)	Serum	1030 cm^−1^ (stretching C-O ribose), 1115 cm^−1^ (RNA), 1373 cm^−1^ (stretching C-N cytosine and guanine), 1431 cm^−1^ (proteins), 1489 cm^−1^ (in-plane CH bending vibration), and 1585 cm^−1^ (amide I).	FS and KNN	When analyzing serum samples for ovarian cancer, the combination yielded a classification rate of 95% ± 8.05%.	Small sample size, the lack of external validation across diverse populations, and potential age-related spectral variability between cancer and control groups.	[31]
	Plasma (*n* = 30) and serum (*n* = 30) samples of patients undergoing surgery for ovarian cancer	Serum and plasma	Stage I vs. stage II–IV: In plasma: around 1323 cm^−1^ and 1350 cm^−1^ (linked to carboxyl and collagen structures) In serum: around 1554 cm^−1^ and 1732 cm^−1^ (related to amide II and lipid carboxyl stretching) Serous vs. non-serous carcinoma: In plasma: 1550 cm^−1^ and 1562 cm^−1^ (C=O stretching in amide II bands) In serum: 1462 cm^−1^ and 1465 cm^−1^ (amide II and CH₂ bending in lipids) Age classification (≤ 60 vs. > 60 years): In plasma: 1173 cm^−1^ and 1188 cm^−1^ (glycogen and lipid-associated stretches) In serum: 1045 cm^−1^ and 1069 cm^−1^ (glycogen and phospholipid/cholesterol esters)	GA-LDA	Using a GA-LDA model with 33 wavenumbers, plasma samples achieved 100% sensitivity and specificity in distinguishing stage I from stages II–IV. For differentiating serous from non-serous types, plasma analysis yielded up to 94% sensitivity and specificity with 29 wavenumbers in the GA-LDA model. When categorizing patients by age (≤60 years and >60 years), plasma samples again reached 100% accuracy using 42 wavenumbers. In serum samples, high diagnostic performance was also observed, with sensitivity and specificity up to 91.6% for stage I vs. stage II–IV, 93.0% for serous vs. non-serous, and 96.0% for age classification.	Small sample size.	[32]
Endometrial cancer	Endometrial cancer cases (*n* = 30) and NCS (*n* = 30)	Plasma	1026 cm^−1^ (glycogen), 1080 cm^−1^ (1080 cm^−1^ (νsPO_2_^−^ related to RNA), 1408 cm^−1^ (COO^−^ symmetric stretching vibrations of fatty acids and amino acid), 1589 cm^−1^ (ring C-C stretch of phenyl), and 1744 cm^−1^ (lipids)	FS-Fisher, KNN, and Identity	The highest classification rate for plasma samples was 77.08% ± 15.87%.	Small sample size, lack of external validation across diverse populations, and potential age-related spectral variability between cancer and control groups.	[31]
	Endometrial cancer cases (*n* = 30) and NCS (*n* = 30)	Serum	1026 cm^−1^ (glycogen), 1080 cm^−1^ (νsPO_2_^−^ ), 1153 cm^−1^, 1466 cm^−1^, 1520 cm^−1^, and 1744 cm^−1^ (lipids)	FS-Fisher, FS-LASSO, FS, and SVM	For serum samples, the ensemble of 3 systems produced a classification rate of 81.67% ± 18.34%.	Small sample size, lack of external validation across diverse populations, and potential age-related spectral variability between cancer and control groups.	[31]
Prostate cancer	17 patients with prostate cancer vs. healthy controls	Saliva	Only small differences at approximately 1500–1650 cm^−1^ and 1000–1100 cm^−1^	PCA-QDA	The optimal PCA-QDA model, using 5 PCs (84.2% explained variance), achieved 93% testing accuracy, 100% clinical sensitivity, and 92% specificity.	The study introduces variability due to uncontrolled hydration levels in saliva samples, which impacts spectral reproducibility and diagnostic accuracy.	[38]
	199 patients with prostate cancer vs. 459 NCS	Serum	~1357 cm^−1^ and ~2947 cm^−1^	NS	The prostate cancer model for male-specific controls had an AUC of 0.86, with 95% sensitivity at 44% specificity and 45% sensitivity at 96% specificity for the sensitivity- and specificity-tuned model, respectively.	Limited methodological clarity regarding preprocessing and the lack of tumor-specific control groups, the absence of genetic tumor information, and the reliance on retrospective data.	[23]
	43 patients with prostate cancer vs. 33 healthy controls	Plasma	Tyrosine (830 and 850 cm^−1^), DNA/RNA (940 cm^−1^, 1085 cm^−1^, 1340 cm^−1^, and 1420 cm^−1^), β-carotene (1160 cm^−1^ and 1525 cm^−1^), amide linkages (1302 cm^−1^ and 1340 cm^−1^), CH_2_ deformation (1340 cm^−1^), tryptophan (~ 1332–1363 cm^−1^), phenylalanine (1006 cm^−1^, 1210 cm^−1^), and lipids and proteins (622 cm^−1^, 644 cm^−1^, 1300 cm^−1^, 1400–1470 cm^−1^, and 1640–1670 cm^−1^)	PLS-DA CLS	A PLS-DA model successfully discriminated between the groups, achieving sensitivity and specificity rates between 90% and 99%. A CLS fitting analysis highlighted key analytes involved in the development and progression of prostate cancer.	Small cohort size, the lack of comprehensive age-matched controls, and the need for validation on larger, clinically diverse datasets.	[37]
Pancreatic cancer	166 patients with pancreatic cancer vs. 459 NCS	Serum	3277 cm^−1^, 1529 cm^−1^, 1636 cm^−1^, 1288 cm^−1^, and 2784 cm^−1^	NS	For pancreatic cancer, the AUC was 0.84, with 95% sensitivity at 42% specificity and 44% sensitivity at 93% specificity for the sensitivity- and specificity-tuned model, respectively.	Limited methodological clarity regarding preprocessing and the lack of tumor-specific control groups, the absence of genetic tumor information, and the reliance on retrospective data.	[23]
	100 pancreatic cancer patients vs. 100 healthy controls vs. 35 symptomatic controls	Serum	~1570 and ~1500 cm^−1^ ~1270 cm^−1^ 1000 and 1100 cm^−1^	RF PLS-DA SVM	Machine learning algorithms were employed, yielding a sensitivity of up to 92% and a specificity of 88% when distinguishing between cancer patients (*n* = 100) and healthy controls (*n* = 100). An ROC analysis produced an AUC of 0.95. Additionally, balanced sensitivity and specificity above 75%, along with an AUC of 0.83, were achieved when comparing cancer cases (*n* = 35) to symptomatic controls (*n* = 35).	Limited by a small sample size and the need for larger prospective trials to validate its clinical applicability.	[21]
CRC	73 CRC patients vs. 44 healthy controls	Serum	Amide I band redshifted by 2 cm^−1^, and amide II band had a significant redshift of 10 cm^−1^	PLS-DA	The AUC for CRC was found to be 0.985.	Limited by potential sample variability, class imbalance, small cohort sizes, and the need for broader clinical validation to confirm diagnostic robustness.	[22]
	200 CRC patients vs. 459 NCS	Serum	1530 cm^−1^, 3351 cm^−1^, 3234 cm^−1^, 1246 cm^−1^, and 1666 cm^−1^	NS	The CRC model demonstrated a strong performance with an AUC of 0.91, achieving 97% sensitivity at 44% specificity and 45% sensitivity at 97% specificity for the sensitivity- and specificity-tuned model, respectively.	Limited methodological clarity regarding preprocessing and the lack of tumor-specific control groups, the absence of genetic tumor information, and the reliance on retrospective data.	[23]
HCC	20 obese cirrhotic patients with HCC vs. 17 without HCC	Plasma	1643 cm^−1^ (amide I) and 1547 cm^−1^ (amide II)	PLS-DA	IR achieved a sensitivity of 0.780 and a specificity of 0.905.	Small sample size and potential overfitting.	[47]
	40 patients with HCC vs. 60 cirrhotic patients	Plasma	1649 cm^−1^, 1639 cm^−1^, and 1547 cm^−1^	Random forest	The FTIR method achieved an AUC-ROC of 0.83, with an accuracy of 0.756, a sensitivity of 0.770, and a specificity of 0.748.	Small sample size, potential artifacts from water subtraction or drying in FTIR spectra, complexity in spectral preprocessing, limited accessibility of advanced instruments, insufficient comparison with established HCC biomarkers, variability in sample handling, and a lack of validation in diverse clinical settings.	[51]
	25 HCC subjects vs. 44 healthy controls	Serum	Amide I band redshifted by 2 cm^−1^	PLS-DA	The AUC was calculated to be 0.987.	Limited by potential sample variability, small cohort sizes, and the need for broader clinical validation to confirm diagnostic robustness.	[22]
	20 patients diagnosed with non-viral HCC vs. 19 age- and gender-matched non-cancer subjects	Serum	Carbohydrate and nucleic acid bands Protein amide I and II bands Lipid CH stretching bands	PCA-LDA	The diagnostic performance of the spectral biomarkers was assessed using multivariate logistic regression and ROC analysis, achieving an AUC of 0.91 (95% CI 0.81–1.0). Notably, these spectral biomarkers outperformed traditional circulating HCC markers, including AFP and PIVKA-II.	This pilot study’s small sample size and potential contamination from the ExoQuick EV isolation method highlight the need for larger cohorts and standardized isolation techniques to confirm its clinical applicability.	[9]
	60 CCA patients, 20 HCC patients, 20 BD patients, and 50 healthy controls	Serum	1284 cm^−1^ (collagen), 1339 cm^−1^, 1035 cm^−1^ (collagen-related bands), 1073 cm^−1^ (phosphate groups), 1152 cm^−1^ (polysaccharides), and 1747 cm^−1^ (lipid ester carbonyl).	PLS-DA, SVM, RF, and NN.	The NN achieved 80–100% sensitivity and 83–100% specificity for differentiating CCA from healthy controls, depending on the spectral region. Combined spectral regions (3000–2800 + 1800–1000 cm^−1^) provided the most accurate results.	The relatively small sample size limited the generalizability of the findings. Due to overlapping spectral signatures, it was difficult to differentiate CCA from HCC and BD.	[53]
Gastric cancer	68 patients with gastric cancers vs. 44 healthy controls	Serum	Amide I band redshifted by 2 cm^−1^	PLS-DA	The AUC was determined to be 0.971.	Limited by potential sample variability, small cohort sizes, and the need for broader clinical validation to confirm diagnostic robustness.	[22]
Oesophageal cancer	*n* = 35 normal, *n* = 18 inflammatory, *n* = 27 Barrett’s, *n* = 6 LGD, *n* = 12 HGD, and *n* = 22 OAC	Plasma	1650 cm^−1^ (amide I) and 1550 cm^−1^ (amide II) 1050–1000 cm^−1^ (carbohydrates and collagen) 1300 cm^−1^ to 1150 cm^−1^ (amide III and vsPO_2_^−^) 1400 cm^−1^, 1260 cm^−1^ (amide III), 1225 cm^−1^ (vsPO_2_^−^), and 1080 cm^−1^ (vsPO_2_^−^)	GA-QDA PCA-QDA	The GA-QDA model achieved perfect classification performance in the prediction set. This model, based on 15 selected wavenumbers, showed excellent sample classification, with only three errors in the training set and one in the validation set. The PCA-QDA model, utilizing seven principal components covering 90% of the data variance, also achieved 100% accuracy, sensitivity, specificity, and F-scores for the normal, inflammatory, Barrett’s, and oesophageal cancer classes. Similarly, the SPA-QDA model demonstrated 100% accuracy in classifying LGD using seven specific variables.	Potential variability in sample drying techniques, limited specificity in some classification models, and the need for validation with larger, more diverse datasets.	[55]
	*n* = 36 normal, *n* = 19 inflammatory, *n* = 28 Barrett’s, *n* = 6 LGD, *n* = 12 HGD, and *n* = 23 OAC	Serum	1000 cm^−1^ to 1338 cm^−1^ (DNA/RNA region), 1435 cm^−1^ to 1573 cm^−1^ (methyl groups of proteins and protein amide II absorption), and 1600 cm^−1^ to 1654 cm^−1^ (amide I)	GA-QDA, SPA-QDA and PCA-QDA	The GA-QDA model, utilizing 13 selected wavenumbers, achieved 100% accuracy, sensitivity, specificity, and F-scores for all OAC stages, with only 4 errors in the training set and 2 in the validation set. The SPA-QDA model, using 7 selected variables, demonstrated sensitivities and specificities above 71% for the normal, inflammatory, Barrett’s, and OAC stages. Similarly, the PCA-QDA models achieved sensitivities and specificities over 71% using seven principal component scores, which accounted for more than 90% of the data variance.	Potential variability in sample drying techniques, limited specificity in some classification models, and the need for validation with larger, more diverse datasets.	[55]
	*n* = 38 normal, *n* = 19 inflammatory, *n* = 27 Barrett’s, *n* = 6 LGD, *n* = 12 HGD, and *n* = 25 OAC	Saliva	1000 cm^−1^ to 1150 cm^−1^ (DNA/RNA region), 1350 cm^−1^ to 1500 cm^−1^ (amide II), and 1530 cm^−1^ to 1600 cm^−1^ (amide I)	PCA-QDA, SPA-QDA, and GA-QDA	The PCA-QDA, SPA-QDA, and GA-QDA models demonstrated excellent classification accuracy, ranging from 88.8% to 100%.	Potential variability in sample drying techniques, limited specificity in some classification models, and the need for validation with larger, more diverse datasets.	[55]
	*n* = 38 normal, *n* = 19 inflammatory, *n* = 27 Barrett’s, *n* = 6 LGD, *n* = 11 HGD, and *n* = 25 OAC	Urine	956 cm^−1^ to 1381 cm^−1^ (DNA/RNA region), 1431 cm^−1^ to 1562 cm^−1^ (amide II region mainly stems from the C–N stretching and C–N–H bending vibrations), and 1681 cm^−1^ to 1777 cm^−1^	PCA-QDA, SPA-QDA and GA-QDA	The PCA-QDA model using urine spectra achieved perfect classification, with 100% accuracy, sensitivity, specificity, and F-scores for test samples based on seven PCA scores. The predicted classification performance for all classes was also 100%. Both the SPA-QDA and GA-QDA models demonstrated high sensitivity across all classes (ranging from 78.2% to 100%) but exhibited low specificity, particularly for normal (SPA-QDA = 35.5% and GA-QDA = 62.5%), HGD (SPA-QDA = 33.3%), and OAC (SPA-QDA and GA-QDA = 20%).	Potential variability in sample drying techniques, limited specificity in some classification models, and the need for validation with larger, more diverse datasets.	[55]
Lung cancer	31 patients with lung cancer vs. 950 healthy controls	Saliva	Small differences at approximately 1500–1650 cm^−1^ and 1000–1100 cm^−1^	PCA-QDA	This PCA-QDA model, built with 8 PCs (90.4% explained variance), acheived 91% testing accuracy, 100% clinical sensitivity, and 91% specificity for the sensitivity- and specificity-tuned model, respectively.	The study introduces variability due to uncontrolled hydration levels in saliva samples, which impacts spectral reproducibility and diagnostic accuracy.	[38]
	201 patients with lung cancer vs. 459 NCS	Serum	1167 cm^−1^, 1074 cm^−1^, 1532 cm^−1^, 2750 cm^−1^, and 1124 cm^−1^	NS	The lung cancer model reached an AUC of 0.91, with perfect sensitivity of 100% at a specificity of 40%, and 47% sensitivity at 95% specificity for the sensitivity- and specificity-tuned model, respectively.	Limited methodological clarity regarding preprocessing and the lack of tumor-specific control groups, the absence of genetic tumor information, and the reliance on retrospective data.	[23]
Brain cancer	247 patients with brain cancer vs. 459 NCS	Serum	1523 cm^−1^, 1607 cm^−1^, 3278 cm^−1^, 2861 cm^−1^, and 1256 cm^−1^	NS	The model achieved an AUC of 0.90, with a sensitivity of 95% at a specificity of 42% and a sensitivity of 46% at a specificity of 99% for the sensitivity- and specificity-tuned model, respectively.	Limited by the lack of tumor-specific control groups, the absence of genetic tumor information, and reliance on retrospective data requiring further prospective validation.	[23]
	90 patients with brain cancer vs. 87 control patients who displayed no symptomatic indications of cancer	Serum	1092.5 cm^−1^, 1003 cm^−1^, 1045.4 cm^−1^, 1083.5 cm^−1^, 1116.5 cm^−1^, 1240.5 cm^−1^, 1075.6 cm^−1^, 1086.5 cm^−1^, 1065 cm^−1^, 1135.5 cm^−1^, 1105 cm^−1^, 1163.5 cm^−1^, 1605.5 cm^−1^, 1640.5 cm^−1^, 1064.5 cm^−1^, 1645.5 cm^−1^, 1635.5 cm^−1^, 1535.6 cm^−1^, and 1540.5 cm^−1^	PLS-DA	For the T1 cohort using the PLS-DA model, the sensitivity was 98.5% (SD: 3.6) with a 95% CI of 97.5–99.5%. The specificity was 95.1% (SD: 3.4) with a 95% CI of 94.2–96.0%, and the balanced accuracy was 96.8% (SD: 2.2) with a 95% CI of 96.2–97.4%. For the T2/FLAIR cohort using the PLS-DA model, the sensitivity was 88.7% (SD: 8.6) with a 95% CI of 86.3–91.1%. The specificity was 94.7% (SD: 3.9) with a 95% CI of 93.6–95.8%, and the balanced accuracy was 91.7% (SD: 4.6) with a 95% CI of 90.4–93.0%.	Reduced discriminatory performance after filtration, reliance on high-molecular-weight components for accurate stratification, and the need for larger cohorts to validate clinical applicability.	[66]
	487 brain tumor samples and 237 healthy controls	Blood serum	1524.5, 1516.5, 1532.5, 1508.5, 1028.5, 1036.5, 1500.5, 1540.5, 1020.5, 1788.5, 1044.5, 1796.5, 1668.5, 1012.5, and 1492.5 cm^−1^	PLS-DA RF SVM	This study achieved a sensitivity of 90.5% and a specificity of 91.1% in distinguishing brain cancer patients from healthy controls using PLS-DA. Although brain tumor prevalence is very low at 1.6%, the PLS-DA model yielded a PPV of 14.2%. Three different machine learning techniques were compared, each demonstrating balanced accuracies around 90%, which would be considered cost-effective.	Limited by the need for further validation with larger cohorts to confirm diagnostic robustness and applicability across diverse clinical settings.	[67]

Abbreviations: IR: infrared; NCS: non-cancerous symptomatic; NS: not specified; AUC: area under curve; DNA: Deoxyribonucleic Acid; RNA: Ribonucleic acid; OPLS-DA: orthogonal partial least squares discriminant analysis; RMSECV: root mean square error of cross-validation; iPCA: improved principal component analysis; MANOVA; Multivariate Analysis of Variance; PCA-DA: principal component analysis discriminant analysis; SVM: support vector machine; PCR: principal component regression; SPA-SVM: successive projection algorithm-support vector machines; FS-LASSO: feature selection-least absolute shrinkage and selection operator; KNN: k-nearest neighbor; GA-LDA: genetic algorithm with linear discriminant analysis; PC: principal components; PLS-DA: partial least squares discriminant analysis; CLS: classification; LDA: linear discriminant analysis; MRMR; Minimum Redundancy and Maximum Relevance; ROC: receiver operating curve; CRC: colorectal cancer; FTIR: Fourier-transform infrared spectroscopy; HCC: hepatocellular carcinoma; GA-QDA: genetic algorithm followed by quadratic discriminant analysis; PCA-QDA: principal component analysis followed by quadratic discriminant analysis; SPA-QDA: successive projection algorithm followed by quadratic discriminant analysis; LGD: low-grade dysplasia; HGD: high-grade dysplasia; OAC: esophageal adenocarcinoma; CI: confidence interval; PPV: positive predictive value; PIVKA-II: protein induced by vitamin K absence or antagonist-II; CCA: cholangiocarcinoma; BD: biliary disease; and NN: neural network.

## 12. Translation to Clinic

Multiple studies have demonstrated the potential of ATR-FTIR spectroscopy in liquid biopsy diagnostics for various solid cancers, with high sensitivity and specificity in many cases. This underscores its versatility and diagnostic accuracy, making it a promising candidate for broad application in cancer diagnostics. Therefore, translating ATR-FTIR spectroscopy into clinical oncology presents a significant potential but necessitates a multifaceted strategy to navigate the scientific, technical, and regulatory challenges for effective implementation.

A fundamental requirement is to establish standardized protocols for sample preparation, spectral acquisition, data preprocessing, and interpretation. Variability in these areas can lead to discrepancies in diagnostic outcomes and compromise reproducibility, which is a key aspect of clinical reliability. Different spectra can be produced by model performance being affected by variations in sample types (e.g., serum versus plasma), drying techniques, and spectral resolution. The creation of a generally accepted benchmark for these attributes is required to guarantee similar outcomes in various healthcare environments [69]. First and foremost, the precision of ATR-FTIR spectroscopy in liquid biopsy diagnosis depends on reliable sample collecting and processing procedures. Spectral and diagnostic accuracy can be significantly impacted by changes in pre-analytical factors, including sample type (plasma vs. serum), collection tubes, anticoagulants, centrifugation rates, and storage conditions [70]. For example, hemolysis, lipemia, and long-term sample storage at improper temperatures might add artifacts that conceal the biofluid’s molecular fingerprint [71]. Furthermore, drying procedures, such as air-drying versus controlled evaporation, affect the uniformity of deposition on the internal reflection element, potentially affecting spectral profiles due to phenomena like the coffee-ring effect [72].

Its clinical application has also been limited owing to several technological hurdles associated with the physics behind the technique rather than the sample itself. A critical component in the spectroscopic analysis of serum samples is the IRE, which must have a refractive index higher than that of the biofluid to generate evanescent waves. Additionally, the IRE is typically fixed to the spectrometer’s top plate, necessitating a step-by-step process where biofluids are individually applied, dried, analyzed, and cleaned. This approach is impractical for fast-paced, high-volume clinical settings. Fixed IRE also presents a risk of biofluid contamination, making it less suitable for clinical environments where a disposable diagnostic platform is preferred [14]. The development of microfabricated silicon slides as IREs has shown significant potential in addressing the practical limitations of ATR-FTIR spectroscopy, paving the way for its clinical application in cancer diagnostics. For example, the Dxcover Infrared Platform features sample slides crafted from microfabricated silicon wafers, replacing the traditional single infrared reflection element with four distinct sampling areas—one for background measurement and three for sample measurements. Additionally, the Dxcover Autosampler automates sample slide analysis by indexing the slide across the infrared beam without requiring user intervention. Orthogonal techniques like SERS and Field-Effect Transistor (FET)-based sensing could enhance ATR-FTIR spectroscopy by providing complementary molecular and electrical insights. While ATR-FTIR spectroscopy detects dipole-driven vibrational modes, SERS amplifies Raman signals, revealing polarizability-based features and enabling ultra-sensitive detection of low-concentration biomarkers. FET sensing measures electrical properties, such as charge density and resistance, offering unique insights into molecular interactions and sample interfaces. This synergy could improve detection accuracy, resolving challenges like peak overlap and enhancing specificity in applications like biomarker discovery [73].

Another key factor in advancing this diagnostic technique for commercial use has been careful consideration of existing challenges in the cancer diagnostic pathway, ensuring that the technology can be effectively implemented for earlier diagnosis while remaining economically viable for healthcare systems. Health economic analyses have demonstrated that the technology is cost-effective as a triage tool in primary or secondary care settings, provided that test sensitivity and specificity are at least 80%. As a result, the technology was recently tested in a prospective clinical validation study at the Western General Hospital in Edinburgh, Scotland, where it achieved a sensitivity of 83.3% and specificity of 87.0% in evaluating symptomatic patients referred to secondary care through open-access CT for brain tumor diagnosis [74]. Thus, ATR-FTIR spectroscopy could offer a cost-effective screening method for symptomatic patients in primary care, potentially stream-lining the current imaging diagnostic pathway, and providing significant cost savings for healthcare services.

As research progresses, establishing repositories of spectral data from patients with cancer, including treatment-naive and post-therapy samples, may be invaluable. These datasets could serve as references for machine learning algorithms, improving their ability to distinguish between cancerous and healthy states as well as different cancer stages or responses to treatment. Furthermore, collaborative networks linking research institutions and hospitals can standardize and pool spectral data, address data scarcity, and enhance model robustness through diverse, representative samples [75].

In summary, integrating ATR-FTIR spectroscopy into clinical cancer diagnostics as a liquid biopsy tool shows promise but requires cautious optimism. While preliminary evidence highlights its potential for detecting and monitoring various cancers, significant technical, logistical, and regulatory challenges remain. To achieve clinical translation, standardized protocols and robust validation through large-scale, multicenter studies are essential. Furthermore, a multi-modal approach that combines ATR-FTIR with other diagnostic tools may enhance its reliability and clinical utility, paving the way for its potential role in oncology.

## Figures and Tables

**Table 1:** Overview of vibrational bands and their corresponding wavenumber ranges (cm^−1^) linked to biomolecules.

Vibrational Band	Wavenumber Range (cm^−1^)	Biomolecules
CH_3_ stretching (symmetric)	~2870–2885 cm^−1^	Lipids
CH_2_ stretching (asymmetric)	~2920–2935 cm^−1^	Lipids
CH_2_ stretching (symmetric)	~2845–2855 cm^−1^	Lipids
Carbonyl (C=O) stretching	~1700–1750 cm^−1^	Proteins
Amide I band	~1600–1700 cm^−1^	Proteins
Amide II band	~1500–1600 cm^−1^	Proteins
CH_2_ scissoring	~1450–1475 cm^−1^	Lipids
Symmetric COO^−^ stretching	~1390–1410 cm^−1^	Proteins
Asymmetric PO_2_^−^ stretching	~1250–1220 cm^−1^	Nucleic acids
Symmetric PO_2_^−^ stretching	~1085–1070 cm^−1^	Nucleic acids

## Data Availability

Not applicable.
